# Supplementary material for: Rashba-splitting-induced topological flat band detected by anomalous resistance oscillations beyond the quantum limit in ZrTe5
Source: Nat Commun. 2024 May 23;15:4407. doi: 10.1038/s41467-024-48761-9 (PMC11116540; doi:10.1038/s41467-024-48761-9)
Supplement: Supplementary file 1 — Supplementary Information [file 41467_2024_48761_MOESM1_ESM.pdf]

Supplementary Information

Rashba-splitting-induced topological flat band detected by anomalous resistance oscillations beyond quantum limit in $ZrTe_5$

Dong Xing[1,2], Bingbing Tong[1], Senyang Pan[3], Zezhi Wang[1,2], Jianlin Luo[1,2], Jinglei Zhang[3], and Cheng-Long Zhang[1*]

[1]*Beijing National Laboratory for Condensed Matter Physics, Institute of Physics, Chinese Academy of Sciences, Beijing 100190, China*

[2]*School of Physical Sciences, University of Chinese Academy of Sciences, Beijing 100049, China.*

[3] *High Magnetic Field Laboratory, HFIPS, Chinese Academy of Sciences, Hefei 230031, China*

Corresponding author: chenglong.zhang@iphy.ac.cn

Supplementary Note 1. Torque data in CVT samples and related symmetry analyses

We also performed the same torque measurement on a sample grown by the chemical vapor transfer (CVT) method. As shown in Supplementary Fig. 3**b**, the angular torque at 7 T displays a negligible asymmetric amplitude reflecting a small A_2 term. By adopting the same fitting process as outlined in the main text, as shown in Supplementary Fig. 3**c**, the ratio $A_2(T)/A_2(250\ K)$ exhibits no enhancement at low temperatures and follows the ratio $A_1(T)/A_1(250\ K)$, which is distinct from the results of Fig. 2**c** in the main text and Supplementary Fig. 3**a**.

We analyze possible crystal structures that flux-grown $ZrTe_5$ samples adopt in low temperatures. Angular torque is powerful for systems with orthogonal axes, like cubic, tetragonal, or orthorhombic structures. Our torque results show the measured results are incompatible with an orthorhombic structure. For a monoclinic structure with a C_2 operation, the χ tensor adopts a form:

$$\chi_{ij} = \begin{pmatrix} \chi_{xx} & \chi_{xy} & 0 \\ \chi_{xy} & \chi_{yy} & 0 \\ 0 & 0 & \chi_{zz} \end{pmatrix}$$

Here ***x, y, z*** are used, and the magnetic torque $\boldsymbol{\tau}$ is:

$$\boldsymbol{\tau} = \frac{1}{2}\mu_0 V H^2 \begin{pmatrix} \sin 2\theta \cdot (\chi_{yy} - \chi_{zz}) \\ \sin 2\theta \cdot (-\chi_{xy}) \\ \sin^2\theta \cdot (2\chi_{xy}) \end{pmatrix}$$

For a triclinic structure with no additional symmetry operations, the χ tensor adopts a general form:

$$\chi_{ij}(C_1) = \begin{pmatrix} \chi_{xx} & \chi_{xy} & \chi_{xz} \\ \chi_{yx} & \chi_{yy} & \chi_{yz} \\ \chi_{zx} & \chi_{zy} & \chi_{zz} \end{pmatrix}$$

and the magnetic torque $\boldsymbol{\tau}$ is:

$$\tau_x = \frac{1}{2}\mu_0 V H^2[(\chi_{yy} - \chi_{zz}) \cdot \sin 2\theta + 2\chi_{yz} \cdot \cos 2\theta]$$

$$\tau_y = \frac{1}{2}\mu_0 V H^2(-\chi_{xy} \cdot \sin 2\theta - 2\chi_{xz} \cdot \cos^2\theta)$$

$$\tau_z = \frac{1}{2}\mu_0 V H^2(\chi_{xz} \cdot \sin 2\theta + 2\chi_{xy} \cdot \sin^2\theta)$$

$ZrTe_5$ adopts a crystal structure with lower symmetry, like monoclinic or triclinic with non-orthogonal axes, complicating the angular magnetic torque measurements and analyses. Nevertheless, in the above analyses, we can see there indeed appears $\sin^2\theta$ term when the magnetic field rotates in the ***yz*** (***bc***) plane, namely the A_2 term, in the χ_{ij} tensor of the two crystal structures with lower symmetries.

Supplementary Note 2. Nonreciprocal transport measured under negligible Joule heating

We performed the nonreciprocal transport measurements on sample S75 with magnetic fields along ***b*** and ***c*** directions, respectively. In the measurements process, we found that the magnetic-field-symmetric Seebeck effect contributes to the raw signal even with a current as low as 0.1 mA. The Seebeck effect is subtracted in the process of field antisymmetrization. While increasing the excitation current, a magnetic-field-asymmetric signal arises at small fields, which superimposes on a linear background after antisymmetrization. The Nernst effect causes this anomaly at a small field due to an unevenly distributed thermal gradient caused by contact resistance Joule heating. We have to set an excitation current of ***i*** = 0.1 mA to minimize this effect. As we know, nonreciprocal resistance[1] adopts a form $R(I_0, B) \propto \gamma R_0 I_0 B$, where γ is the coefficient that characterizes the strength of nonreciprocal resistance. We adopted 2 ω measurements[2,3], which produces $V_{2\omega} = \frac{1}{2}\gamma R_0 B I_0^2$, exhibiting a linear-in-B second-harmonic voltage. As shown in Supplementary Fig. 4**a**, we now can recover this linear-

in-B second-harmonic voltage and find that the nonreciprocal signal $V_{xx}^{2\omega}$ (**B**//$\boldsymbol{c}$) is larger than $V_{xx}^{2\omega}$ (**B**//$\boldsymbol{b}$). This means there is a polar component (**P**) along the out-of-plane ***b*** axis in our samples, unlike the negligible polarity along the ***b*** axis in ref[4].

Supplementary Note 3. Angle-dependent $\boldsymbol{\rho_{yx}}$ for *ba* and *bc* planes

As shown in Supplementary Fig. 5**a** and 5**b**, we measured angle-dependent Hall resistivity ρ_{yx} in both ***ba*** and ***bc*** planes, respectively. There is no in-plane anomalous Hall (AHE) signal in our flux-grown samples. Our samples are different from those used in ref[5], but similar to those used in ref[6]. Our results are also consistent with the angular ρ_{yx} reported in ref[6]. Therefore, the AHE appears when the magnetic field is along ***b*** axis, and suddenly disappears when the magnetic field is tilted along ***ac*** in-plane configuration, supporting the Rashba-splitting picture proposed in the main text.

Supplementary Note 4. Process of background subtraction for obtaining the oscillatory $\boldsymbol{\Delta\rho_{xx}}$

As shown in Supplementary Fig. 6, the magnetoresistance increases very quickly at small fields and tends to saturate at higher fields, which is found to be well fitted by exponential functions $c * e^{-(x-x_0)/t_1}$, which is a smooth function shown by the black fitting line.

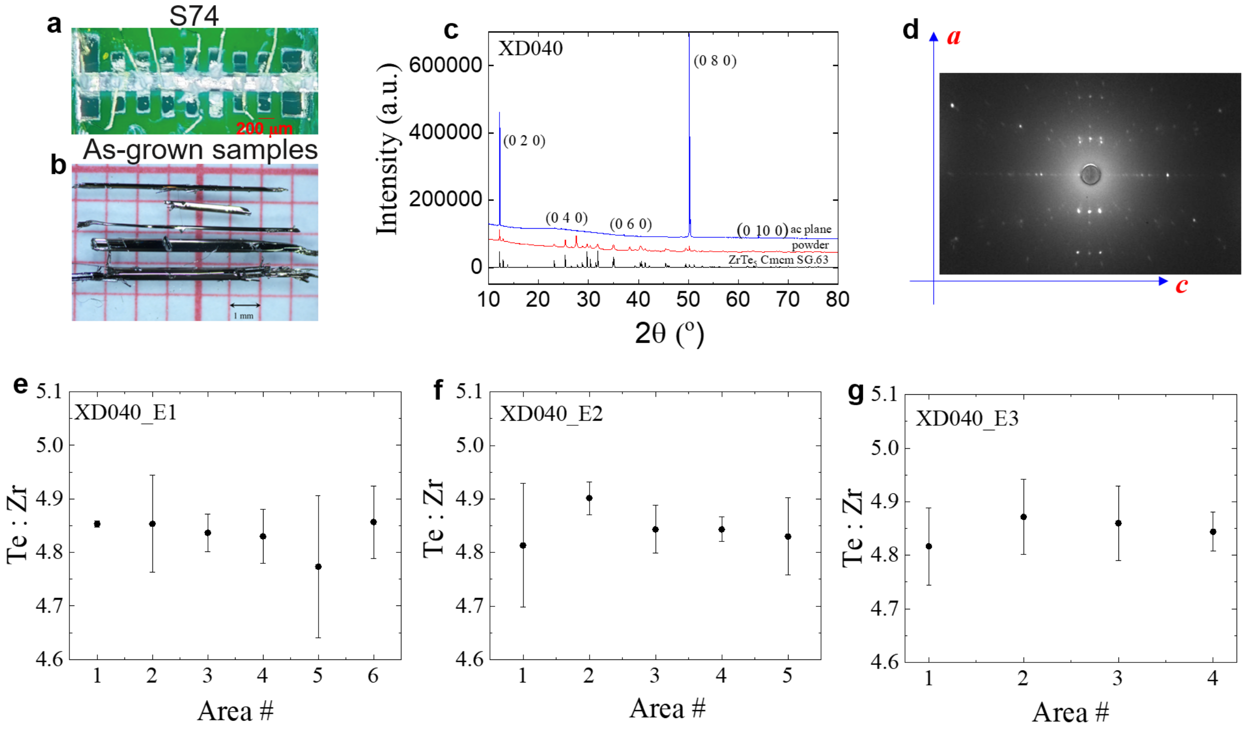

Supplementary Fig. 1: Structural characterizations of $ZrTe_5$. a, The transport device of sample S74. **b**, As-grown single crystals of the batch used in our work. **c**, X-ray diffraction structural characterizations, **d**, Laue image on ***ac*** plane. **e-g**, Energy dispersive x-ray (EDX) composition mapping on three samples.

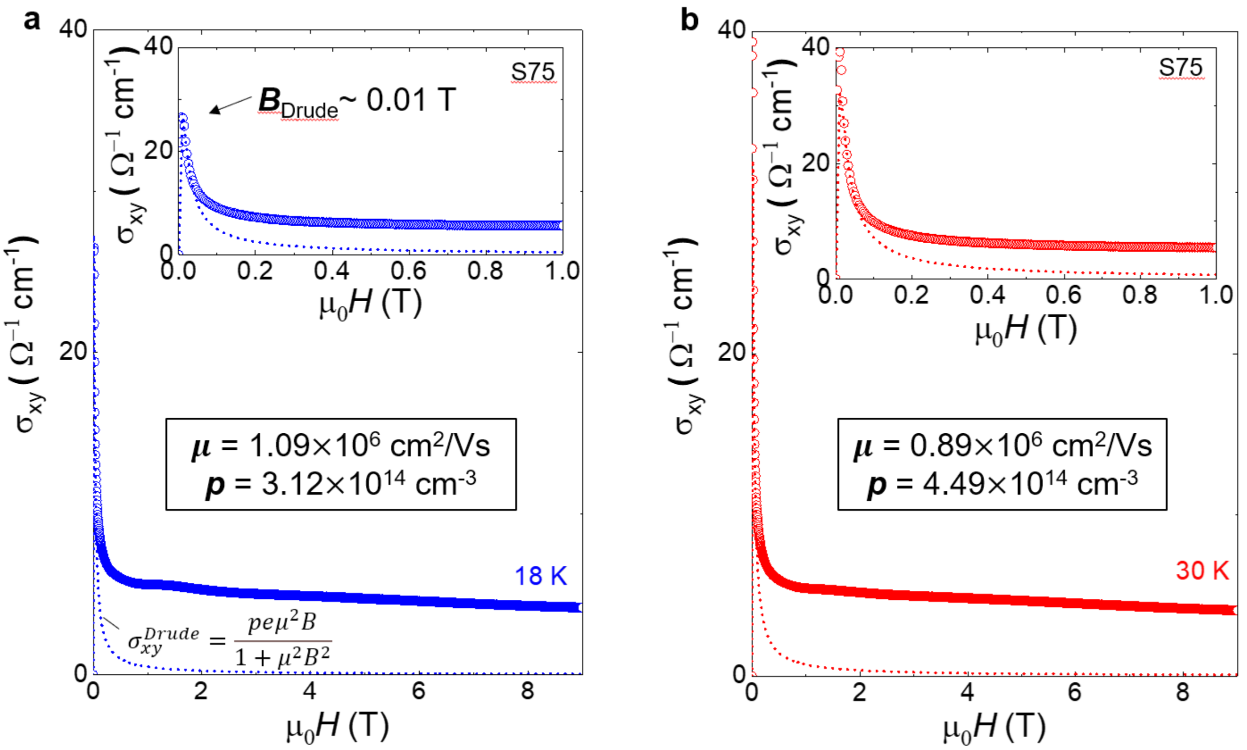

Supplementary Fig. 2: Drude fittings to Hall conductivity of sample S75. a, Hall conductivity measured at 18 K, fitted by the Drude model indicated by the blue dashed line. The inset shows the resonant mobility peak with a characteristic magnetic field ~ 0.01 T. **b**, Hall conductivity measured at 30 K, fitted by the Drude model indicated by the red dashed line. The inset shows the resonant mobility peak. Raw data are reproduced from Fig. 1**d.**

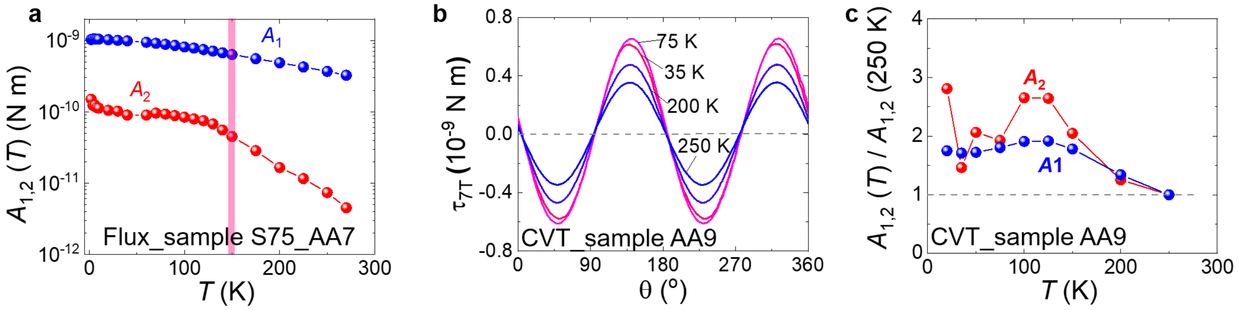

Supplementary Fig. 3: Raw data of Fig. 2c and magnetic torque measurements in CVT-grown sample. a, Raw data of A_1 and A_2 for Fig. 2**c** in the main text, showing the A_2 term suddenly appears around T =150 K, while A_1 is smooth and almost unchanged in the whole temperature range. **b**, Angle-dependent magnetic torque measured on a CVT-grown sample. **c**, Ratios of $A_{1,2}(T)/A_{1,2}(250\ K)$ for CVT-grown sample, showing no anomaly on A_2, which also follows the temperature dependence of A_1.

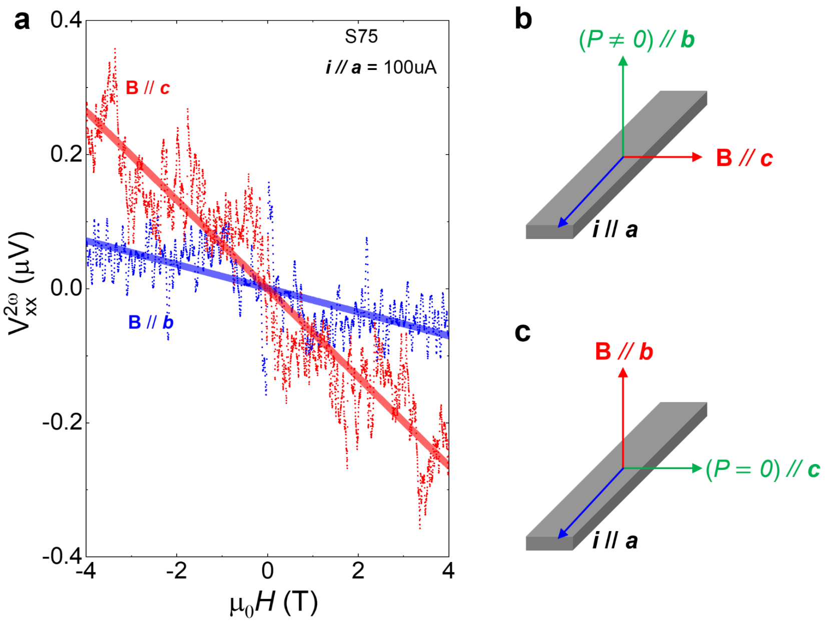

Supplementary Fig. 4: Nonreciprocal resistance measurement in sample S75. a, Second harmonic voltage $V_{xx}^{2\omega}$ for **B**//***c*** and **B**//***b***, respectively. **b** & **c**, Appearance of linear-in-B $V_{xx}^{2\omega}$ for the magnetic field along ***c*** axis indicates there is a polarity component along ***b*** axis.

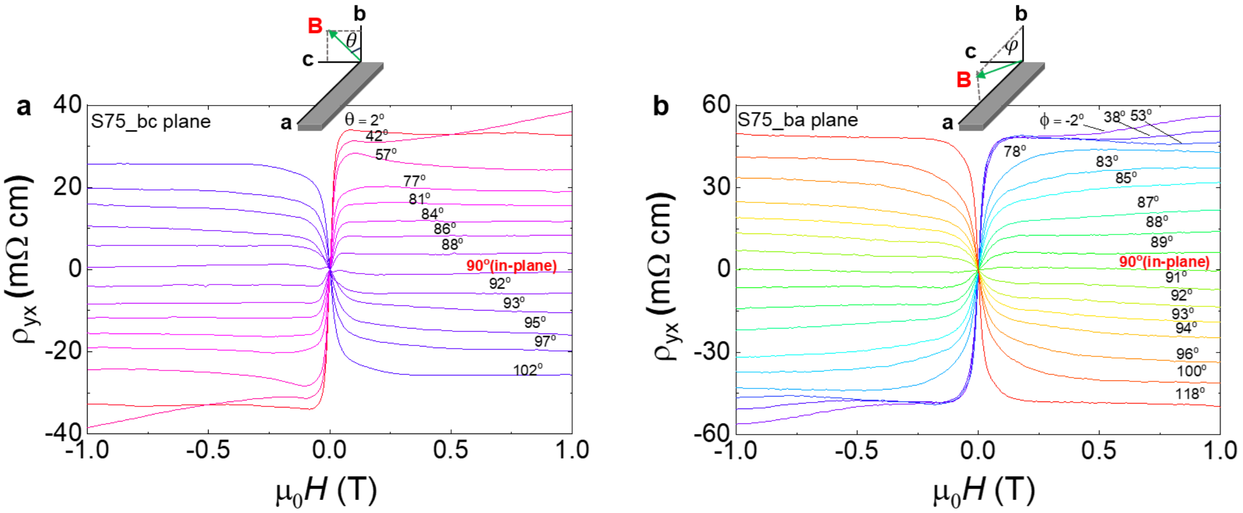

Supplementary Fig. 5: Angle dependence of ρ_{yx} in *bc* and *ba* planes for sample S75. **a**, ρ_{yx} measured in ***bc*** plane with rotating angle θ (inset). $\theta = 90^o$ corresponds to the magnetic field along **c** axis. **b**, ρ_{yx} measured in ***ba*** plane with rotating angle ϕ (inset). $\phi = 90^o$ corresponds to the magnetic field along **a** axis.

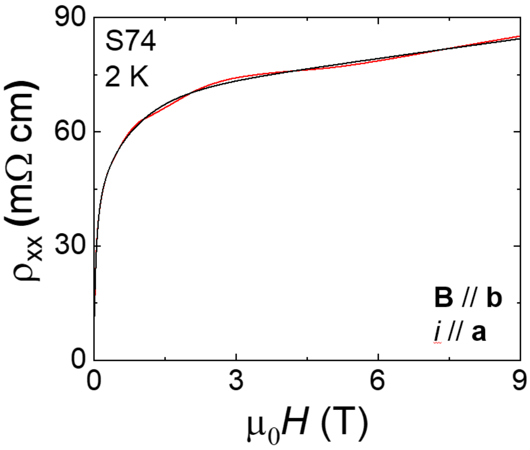

Supplementary Fig. 6: Background subtraction for obtaining the oscillatory $\Delta\rho_{xx}$.

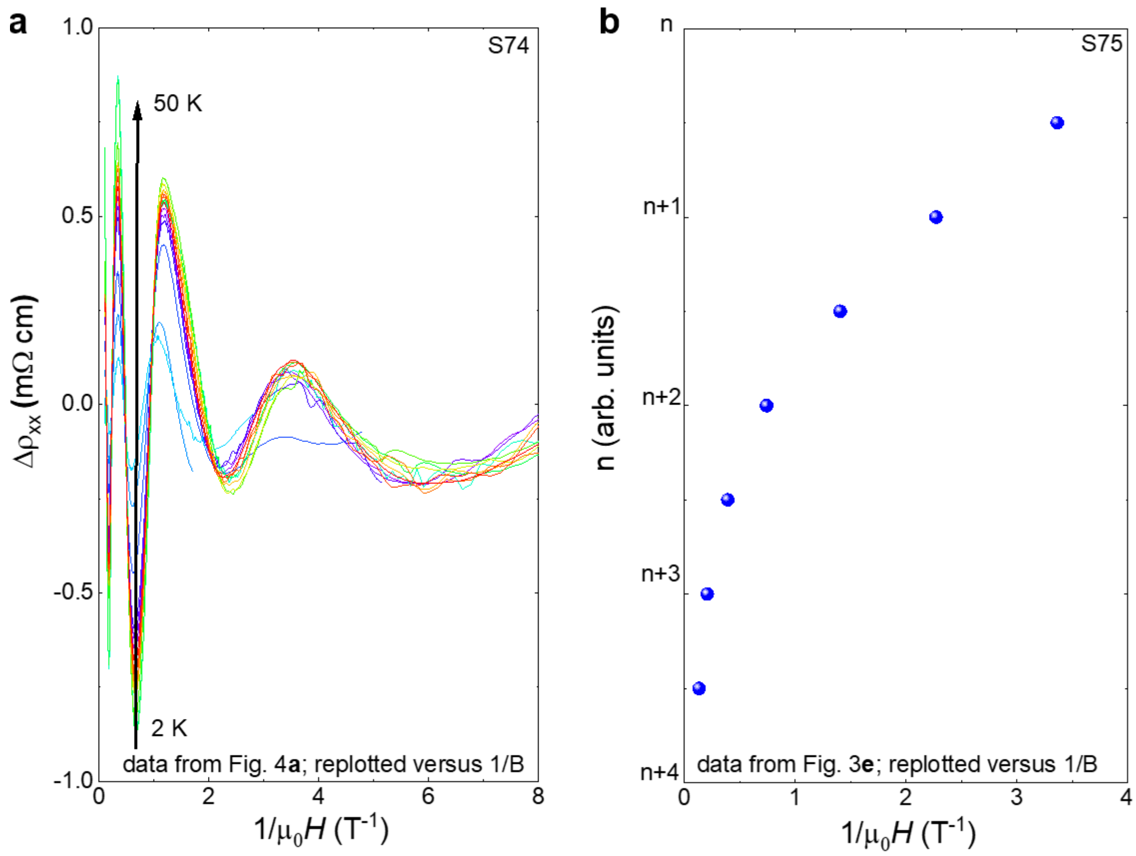

Supplementary Fig. 7: Quantum oscillations in a standard 1/B plot. a, The quantum oscillations replotted in 1/B. **b**, Landau fan diagram. The raw data are reproduced from Fig. 3**e** and 4**a** of the main text.

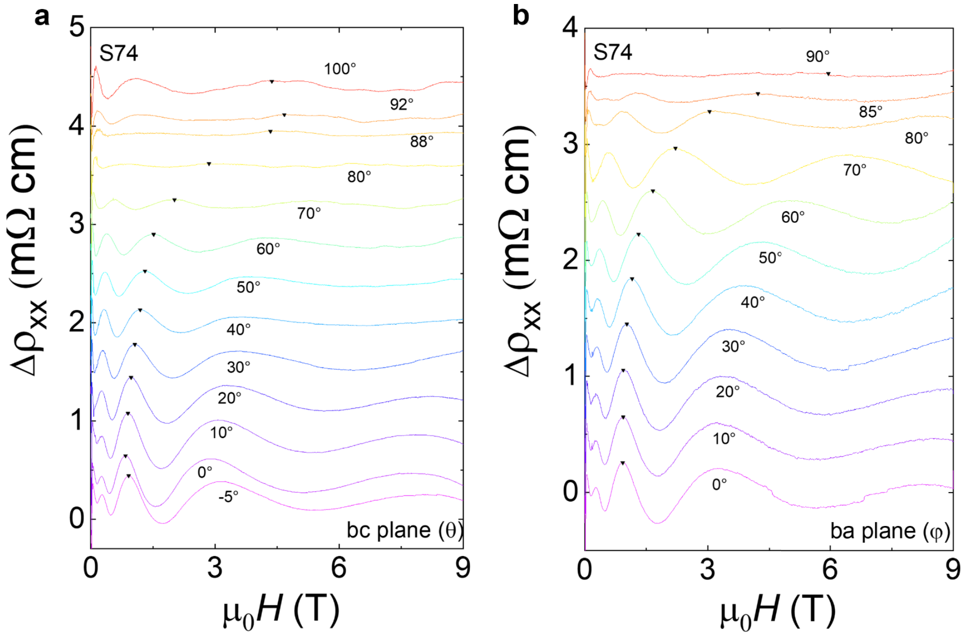

Supplementary Fig. 8: Angle dependence of $\Delta\rho_{xx}$ for sample S74. a, Angle-dependent oscillatory $\Delta\rho_{xx}$ when the magnetic field is rotated within ***bc*** plane. **b**, Angle-dependent oscillatory $\Delta\rho_{xx}$ when the magnetic field is rotated within ***ba*** plane.

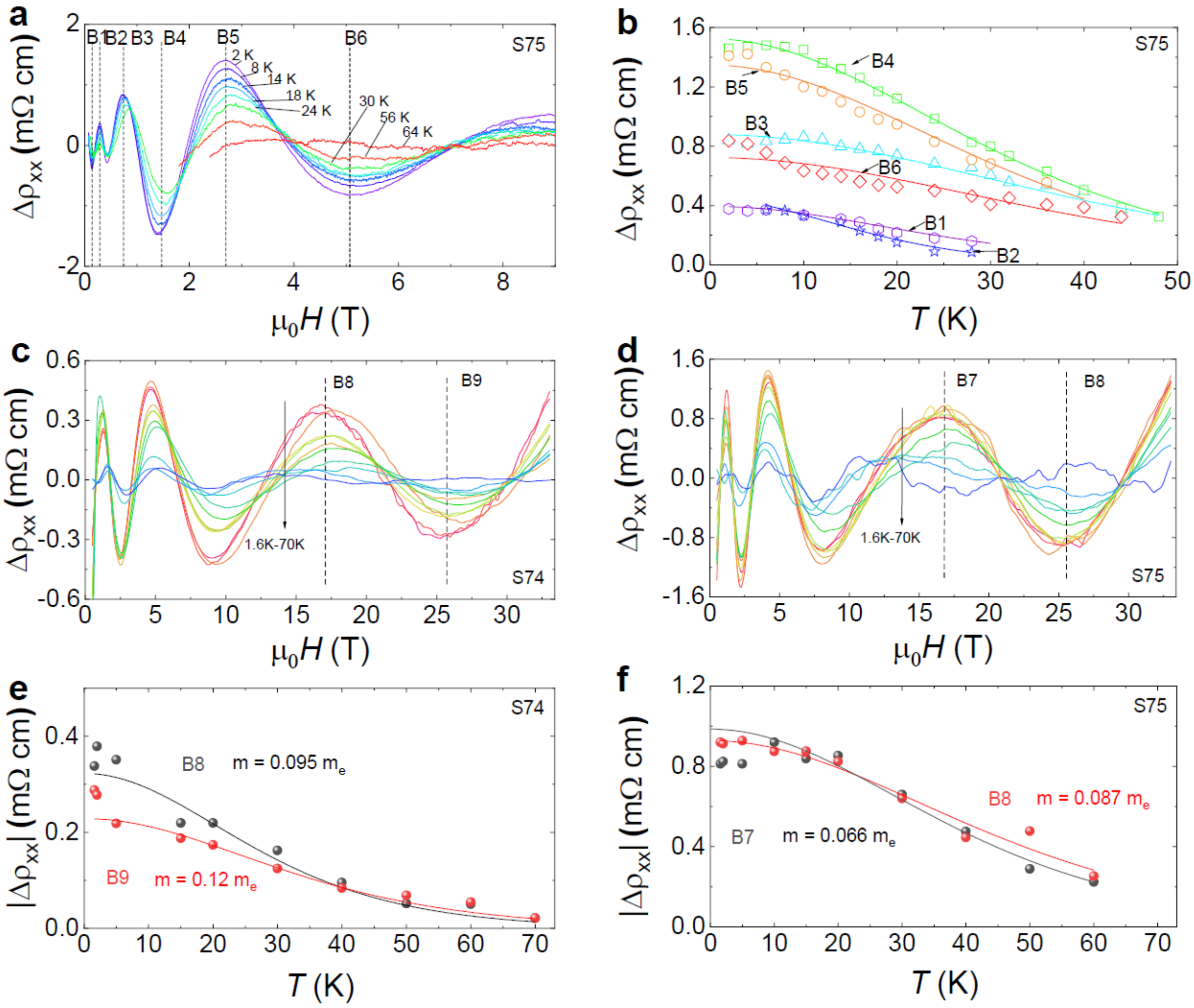

Supplementary Fig. 9: Additional data of temperature-dependent $\Delta\rho_{xx}$. a, Temperature-dependent $\Delta\rho_{xx}$ of S75. **b**, The L–K formula fittings for data from **a**. **c** & **d**, Temperature-dependent $\Delta\rho_{xx}$ of samples S74 and S75 measured in high fields. **e & f**, The L–K formula fittings for high-field oscillations. Data in Supplementary Fig. 9**e** is reproduced from the inset of Fig. 4**b**.

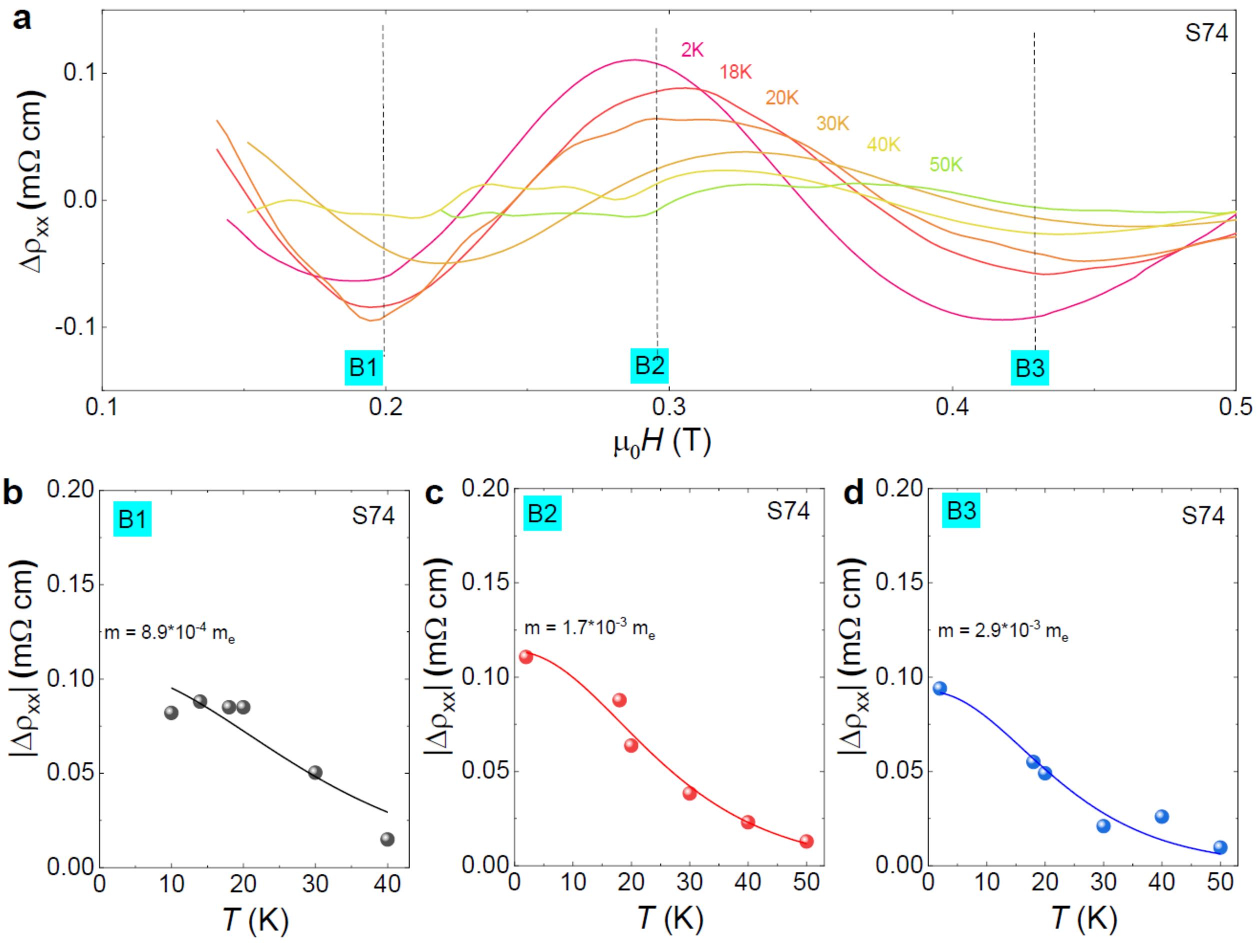

Supplementary Fig. 10: Temperature dependence of low-field oscillations in S74. a, Temperature-dependent $\Delta\rho_{xx}$ (smoothed) of S74 below 0.5 T. **b-d**, The L–K formula fittings for B1, B2 and B3.

Supplementary References

1 Tokura, Y. & Nagaosa, N. Nonreciprocal responses from non-centrosymmetric quantum materials. *Nat. Commun.* **9**, 3740 (2018).

2 Ideue, T. *et al.* Bulk rectification effect in a polar semiconductor. *Nat. Phys.* **13**, 578-583 (2017).

3 Zhang, C.-L. *et al*. Giant Berry curvature dipole density in a ferroelectric Weyl semimetal. *npj Quantum Materials* **7**, 103 (2022).

4 Wang, Y. *et al.* Gigantic magnetochiral anisotropy in the topological semimetal $ZrTe_5$. *Phys. Rev. Lett.* **128**, 176602 (2022).

5 Liang, T. *et al.* Anomalous hall effect in $ZrTe_5$. *Nat. Phys.* **14**, 451-455 (2018).

6 Mutch, J. *et al*. Abrupt switching of the anomalous Hall effect by field-rotation in nonmagnetic $ZrTe_5$. *arXiv preprint arXiv:2101.02681* (2021).
